# Assessment of Optical Quality at Different Contrast Levels in Pseudophakic Eyes

**DOI:** 10.1155/2016/4247973

**Published:** 2016-01-28

**Authors:** Chang Won Park, Hyojin Kim, Choun-Ki Joo

**Affiliations:** ^1^Department of Ophthalmology and Institute for Visual Science, College of Medicine, The Catholic University of Korea, Seoul 06591, Republic of Korea; ^2^Department of Visual Optics, Division of Health Science and Graduate School of Health and Welfare, Baekseok University, Cheonan 31065, Republic of Korea

## Abstract

*Purpose*. To assess visual function using Optical Quality Analysis System (OQAS) at varying levels of contrast in pseudophakic eyes.* Methods*. The study included patients admitted to Seoul St. Mary's Hospital between January and February 2012: 143 pseudophakic eyes with one of five intraocular lens types, examined 2–6 months after cataract surgery, and 93 normal eyes (enhanced visual acuity (VA) < 0.1 logMAR) in age-matched controls. Subjects were examined at three contrast levels using the OQAS.* Results*. At 100%, 20%, and 9% contrast, simulated mean VA was 0.16 ± 0.18 logMAR, 0.30 ± 0.18 logMAR, and 0.52 ± 0.17 logMAR, in normal eyes, and 0.16 ± 0.12 logMAR, 0.33 ± 0.20 logMAR, and 0.56 ± 0.21 logMAR, respectively, in pseudophakic eyes. Simulated VA decreased significantly when contrast was reduced, regardless of ocular status, age group, and lens type (*p* < 0.05). There were no significant differences between normal and pseudophakic eyes among subjects aged 50–69 (*p* > 0.05). Among subjects aged 70–79, pseudophakic eyes showed improved simulated VA (*p* = 0.000) and objective scattering index values (*p* = 0.008).* Conclusions*. Patients with intraocular lenses have similar or superior visual function when compared to those with normal eyes at 2–6 months after cataract surgery, even under low-contrast conditions.

## 1. Introduction

A cataract increases lens opacity and reduces visual acuity (VA), thus impairing the patient's quality of life [1]. The condition may even lead to blindness [2–4]. Surgical treatment is therefore necessary. Various techniques for cataract surgery have been developed since H. Ridley introduced intraocular lenses (IOLs) composed of polymethylmethacrylate in 1949 [5]. The stability of IOLs allows cataract surgery to be commonly performed worldwide, and technological advancements such as multifocal and toric IOLs have increased the procedure's popularity [6, 7]. To improve quality of life, cataract surgery is also performed for correcting refractive error [8–10].

In previous studies, postoperative VA, contrast sensitivity, and optical aberrations were measured as objective indices of surgical success [11–16]. After cataract surgery, improvement in VA is typically tested with a high-contrast (100%) chart under photopic and mesopic conditions [17]. However, the level of contrast in the actual optical environment varies greatly [18]. For example, visual inspection of human faces—of the utmost importance in daily life—involves a target of large size and low contrast. The change in VA after cataract surgery has not been studied under various levels of contrast. The aim of this study was to assess the visual performance of pseudophakic eyes after cataract surgery at different contrast levels by using the OQAS (Optical Quality Analysis System, Visiometrics, Terrassa, Spain).

## 2. Materials and Methods

This research was approved by the Institutional Review Board of the Catholic Medical Center at the Catholic University of Korea and conducted in accordance with ethical research guidelines. The present study adhered to the Declaration of Helsinki and was approved by the Institutional Review Board of the Catholic Medical Center at the Catholic University of Korea (approval number: KC12RISI0023). Patients completed an informed consent form approved by the institutional review board after the purpose of the study was explained to them. The study included patients admitted at Seoul St. Mary's Hospital between January and February 2012. No patients had a history of ocular surgery, ocular disease, or general disorders affecting vision (e.g., diabetic retinopathy) [19]. Vision of patients greater than 0.1 logMAR was measured using the Snellen test and classified as age-matched (50s, 60s, and 70s) between normal and pseudophakic eyes. All patients underwent a comprehensive ophthalmologic examination, slit-lamp evaluation. The patients with pseudophakic eyes had undergone phacoemulsification and received one of five IOL types in the posterior chamber 2–6 months before the study. Patients were stratified into the following age groups: 50–59 years, 60–69 years, and 70–79 years.

Patients with failed IOL implantation into the lens capsule, severe posterior lens capsule opacification or history of laser capsulotomy due to opacification, or any other eye complication were excluded. Those with poor cooperation were also excluded. To prevent uncorrected refractive error from limiting contrast sensitivity or VA [20, 21], trial lenses were worn throughout testing. VA was measured using a Snellen chart at 6 m. Altered contrast sensitivity was simulated with the OQAS by using the double-pass technique. With this approach, the retinal image, degree of haze inside the eye, and condition of visual function are analyzed in terms of objective scattering index (OSI), modulation transfer function (MTF) cut-off value, and Strehl ratio, respectively [22–25]. Simulated VA by OQAS was evaluated at contrast of 100%, 20%, and 9%.

Testing at each contrast level was performed for all five types of IOLs: HOYA PC-60AD (HOYA, Corp, Tokyo, Japan), EC-1PAL (Aaren Scientific, Ontario, Canada), Akreos MI-60 (Bausch & Lomb, Rochester, NY, USA), NY-60 (HOYA, Corp, Tokyo, Japan), and XL Stabi ZO (Carl Zeiss Meditec, Jena, Germany).

One skilled tester (CWP) conducted all the measurements. The average values of three repeated measurements were analyzed. Analysis of variance (ANOVA) and independent-sample* t*-tests were performed using SPSS version 18.0 software (IBM Corporation, Armonk, NY). Values of *p* < 0.05 were considered significant.

## 3. Results and Discussion

The subjects' characteristics are shown in Table 1. Mean visual acuities for the normal eyes were 0.16 ± 0.18 logMAR, 0.30 ± 0.18 logMAR, and 0.52 ± 0.17 logMAR at 100%, 20%, and 9% contrast, respectively (Table 2). In normal eyes from all age groups, simulated VA decreased significantly when contrast was reduced (50–59, *p* = 0.000; 60–69, *p* = 0.000; 70–79, *p* = 0.020). However, simulated VA was highest among those aged 50–59 and lowest among those aged 70–79. Simulated VA at 100% and 9% contrast decreased with increasing age (100%, *p* = 0.045; 9%, *p* = 0.010). No significant differences were noted among the age groups at 20% contrast (*p* = 0.070).

For pseudophakic eyes, mean visual acuities were 0.16 ± 0.12 logMAR, 0.33 ± 0.20 logMAR, and 0.56 ± 0.21 logMAR at 100%, 20%, and 9% contrast, respectively (Table 3). As in the normal eyes, VA decreased significantly when contrast was reduced (*p* = 0.000). However, nova was similar in all age groups (*p* > 0.05).

**Figure 1:**
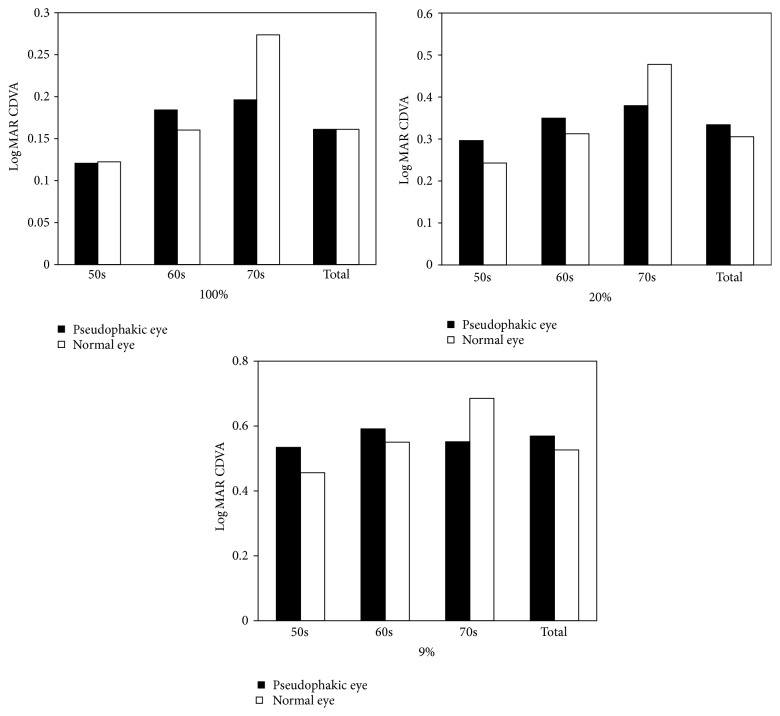
Simulated visual acuity (mean VA logMAR ± SD) of normal versus pseudophakic eyes at various simulated contrast levels, compared by age group.

Figure 1 shows simulated VA for the normal and pseudophakic eyes in each age group at all contrast levels. No significant difference in VA was noted among those aged 50–69. However, among those aged 70–79, the pseudophakic eyes exhibited significantly higher VA (*p* = 0.000).

Table 4 shows the simulated mean visual acuities of the pseudophakic eyes according to IOL type. Subjects implanted with EC-1PAL and NY-60 IOLs showed the lowest and the highest VA, respectively, although no significant differences were noted among IOLs at any contrast level. Regardless of IOL type, VA decreased significantly when contrast was reduced (*p* = 0.000).

**Figure 2:**
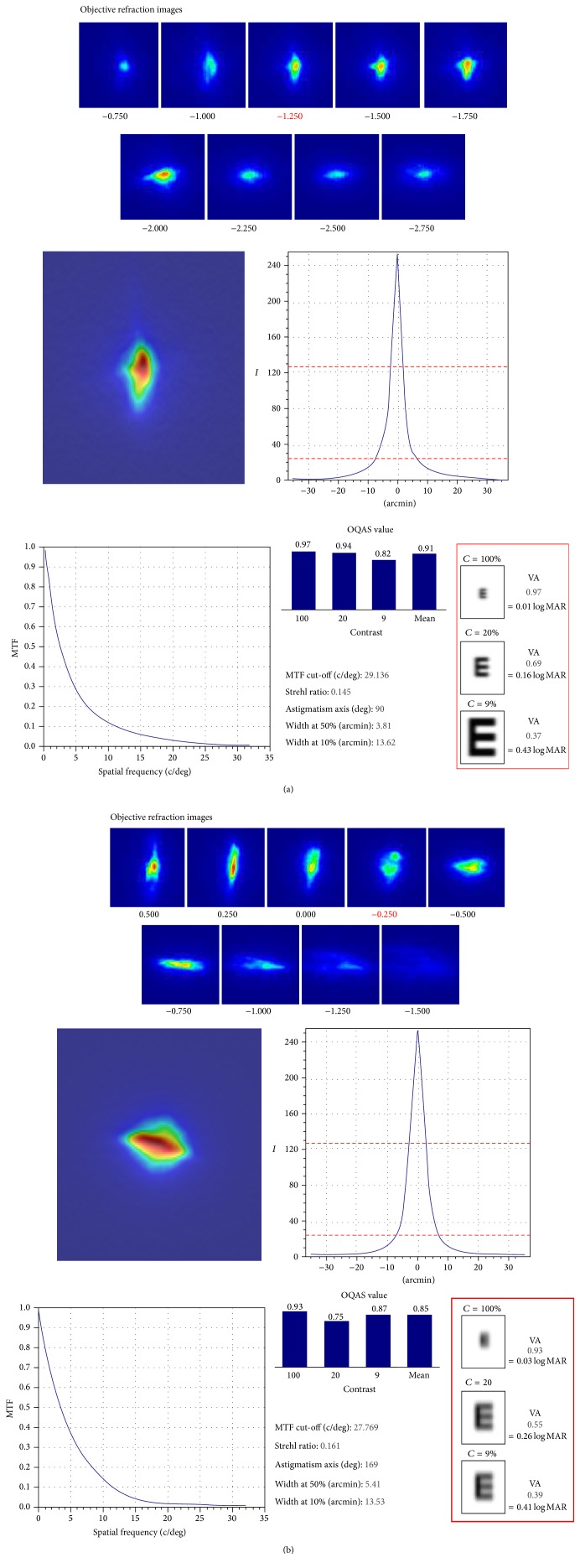
Representative OQAS results of (a) normal and (b) pseudophakic eyes. The red rectangles show Sim VA (simulated visual acuity) at different simulated contrast levels.

OSI value, MTF cut-off value, and Strehl ratio values are presented in Table 5. The mean values were 2.21 ± 1.38, 23.29 ± 10.72, and 0.18 ± 0.47, respectively, in the pseudophakic group and 1.99 ± 1.41, 23.41 ± 9.72, and 0.13 ± 0.07, respectively, in the normal group. The only significant finding was elevated OSI values in normal eyes from the 70–79 age group (*p* = 0.008).  Figure 2 shows representative OQAS results obtained from a 69-year-old patient with normal eyes and a 72-year-old patient with pseudophakic eyes.

In addition to questionnaires, VA and contrast sensitivity assessments are used to evaluate ocular health after cataract surgery. Recent technological advancements have allowed for the measurement of higher-order aberrations including spherical aberration and coma aberration [26] as well as the optical analysis of light spread within the eye (point spread function) [27–29]. However, such tests are useful only in characterizing symptoms [30]. Anera et al. [31] and Artal et al. [32] used the OQAS to measure the OSI, MTF cut-off value, and Strehl ratio after cataract and refractive surgeries and to assess visual function objectively. The OQAS can also be used to simulate contrast changes. In this study, we investigated vision at three contrast levels simulated using the OQAS and stratified subjects with normal and pseudophakic eyes by age.

Among patients in their 50s and 60s, normal eyes exhibited Sim VA superior or similar to that of pseudophakic eyes. However, among those in their 70s, pseudophakic eyes had significantly superior VA (*p* = 0.000). Cataract surgery may have restored visual function in these patients. Alternatively, the normal eyes in this age group may have been free of any lens opacity that could be detected by slit-lamp examination but may have had fine opacities in the lens and/or vitreous chamber that scattered light, thereby reducing VA. Another significant finding was the lower OSI value of the pseudophakic eyes in this group (*p* = 0.008), which is in line with the report by Saad et al. [33] that OSI values increase with age in normal eyes.

In both the normal and pseudophakic groups, VA was directly related to contrast level regardless of age. Furthermore, VA decreased as age increased; as reported by Mathai et al. [34] Sim VA was similar in normal and pseudophakic eyes of subjects aged 50–69 but superior in the pseudophakic eyes of subjects aged 70–79 years. Therefore, visual function must have returned to normal levels in all patients with IOLs at 2–6 months after cataract surgery.

The NY-60 and EC-1PAL IOLs were associated with the highest and lowest visual acuities, respectively, regardless of contrast level. Interestingly, use of the NY-60 IOL is reportedly associated with fewer capsular folds than use of a three-piece IOL [35]. NY-60 IOL users also report the absence of entoptic phenomena [36] 1 year after surgery. In an eye with an IOL, the presence of entoptic phenomena correlates directly with the OSI value [37]. This relationship may explain the superior VA of the NY-60 group, although this improvement in VA was associated with use of the AcrySof SN60WF and TECNIS ZCB00 IOLs in previous reports [38]. In this study, reduced contrast was associated with decreased acuity across groups, but no significant difference in simulated VA among IOL types was noted at any contrast level (*p* = 0.413).

A possible limitation of our study is that the OQAS's He-Ne diode laser may have introduced error to the OSI readings, for example, in patients with dry eye symptoms or cloudy vitreous, which scatters light. High scatter increases OSI values.

## 4. Conclusion

In summary, VA at 2–6 months after cataract surgery in pseudophakic eyes is similar to that of normal eyes and older patients with pseudophakic eyes have superior simulated VA to age-matched controls, contrary to previous reports that the level of contrast and age affect visual function. These findings suggest that IOLs ensure simulated VA similar to that of the normal eye even in very low-contrast conditions, as encountered when driving at night. Therefore, IOL implantation should have a beneficial impact on the patient's quality of life. The OQAS seems to be a useful instrument for the objective evaluation of visual quality under contrast after various surgeries such as cataract surgery, laser-assisted in situ keratomileusis (LASIK), laser-assisted subepithelial keratectomy (LASEK), and keratoconus surgery.

## Figures and Tables

**Table 1:** Characteristics of normal (*N* = 93) and pseudophakic (*N* = 143) eyes.

Characteristic	Normal group	Pseudophakic group
Eyes per age group (*n*)		
50s (M/F)	36 (17, 19)	54 (22, 32)
60s (M/F)	45 (25, 20)	77 (30, 47)
70s (M/F)	12 (6, 6)	12 (5, 7)
Age (years)	62.39 ± 6.60	63.21 ± 6.74
Total gender	48/45	57/86

Values are presented as mean ± SD.

**Table 2:** Simulated visual acuity (mean VA logMAR ± SD) of normal eyes (*N* = 93) at different simulated contrast levels compared by age group.

Age group	Contrast			*p* ^*∗*^
	100%	20%	9%	
50s	0.12 ± 0.11	0.24 ± 0.15	0.45 ± 0.14	0.000
60s	0.16 ± 0.18	0.31 ± 0.19	0.55 ± 0.18	0.000
70s	0.27 ± 0.20	0.47 ± 0.20	0.68 ± 0.16	0.020
Total	0.16 ± 0.18	0.30 ± 0.18	0.52 ± 0.17	0.000

*p* ^*∗∗*^	0.045	0.070	0.010	

^*∗*^ANOVA among contrast levels.

^*∗∗*^ANOVA among age groups.

**Table 3:** Simulated visual acuity (mean VA logMAR ± SD) of pseudophakic eyes (*N* = 143) at different simulated contrast levels, compared by age group.

Age group	Contrast			*p* ^*∗*^
	100%	20%	9%	
50s	0.12 ± 0.16	0.29 ± 0.24	0.53 ± 0.21	0.000
60s	0.18 ± 0.15	0.35 ± 0.23	0.59 ± 0.22	0.000
70s	0.19 ± 0.10	0.37 ± 0.14	0.55 ± 0.19	0.000
Total	0.16 ± 0.12	0.33 ± 0.20	0.56 ± 0.21	0.000

*p* ^*∗∗*^	0.269	0.263	0.332	

^*∗*^ANOVA among contrast levels.

^*∗∗*^ANOVA among age groups.

**Table 4:** Simulated visual acuity (mean VA logMAR ± SD) of pseudophakic eyes (*N* = 143) at different contrast levels, compared by intraocular lens type.

Intraocular lens (*n*)	Contrast			*p* ^**∗**^
	100%	20%	9%	
HOYA PC-60AD (42)	0.17 ± 0.25	0.33 ± 0.26	0.57 ± 0.23	0.000
EC-1PAL (36)	0.18 ± 0.19	0.34 ± 0.18	0.57 ± 0.16	0.000
Akreos MI-60 (25)	0.14 ± 0.27	0.34 ± 0.25	0.58 ± 0.22	0.000
NY-60 (21)	0.09 ± 0.16	0.27 ± 0.18	0.53 ± 0.19	0.000
XL Stabi ZO (19)	0.19 ± 0.22	0.33 ± 0.20	0.56 ± 0.18	0.000

*p* ^*∗∗*^	0.413	0.684	0.870	

^*∗*^ANOVA among contrast levels.

^*∗∗*^ANOVA among lens types.

**Table 5:** OQAS parameters (mean ± SD) of normal (*N* = 93) and pseudophakic (*N* = 143) eyes, stratified by age group.

Parameter	Age group	Normal group	Pseudophakic group	*p* ^*∗*^
OSI (OSI value)	50s	1.82 ± 1.19	2.05 ± 1.28	0.625
	60s	1.92 ± 1.11	2.32 ± 1.52	0.329
	70s	3.96 ± 2.29	2.15 ± 1.31	0.008
	Total	1.99 ± 1.41	2.21 ± 1.38	0.940

MTF cut-off value (C/deg)	50s	22.29 ± 7.88	25.73 ± 11.38	0.273
	60s	23.09 ± 10.72	22.36 ± 10.93	0.773
	70s	15.83 ± 9.14	22.04 ± 9.48	0.090
	Total	23.41 ± 9.72	23.29 ± 10.72	0.925

Strehl ratio	50s	0.13 ± 0.07	0.15 ± 0.15	0.356
	60s	0.12 ± 0.05	0.14 ± 0.13	0.452
	70s	0.10 ± 0.03	0.13 ± 0.03	0.442
	Total	0.13 ± 0.07	0.18 ± 0.47	0.498

^*∗*^ *t*-test.

OSI: objective scattering index; MTF: modulation transfer function.
